# Urinary neutrophil gelatinase-associated lipocalin identifies critically ill young children with acute kidney injury following intensive care admission: a prospective cohort study

**DOI:** 10.1186/s13054-015-0910-0

**Published:** 2015-04-21

**Authors:** Alexandra JM Zwiers, Saskia N de Wildt, Joost van Rosmalen, Yolanda B de Rijke, Erik AB Buijs, Dick Tibboel, Karlien Cransberg

**Affiliations:** Intensive Care and Department of Pediatric Surgery, Wytemaweg 80, 3015 CN Rotterdam, the Netherlands; Department of Pediatric Nephrology, Erasmus Medical Center–Sophia Children’s Hospital, Wytemaweg 80, 3015 CN Rotterdam, the Netherlands; Department of Biostatistics, Erasmus Medical Center, Wytemaweg 80, 3015 CN Rotterdam, the Netherlands; Department of Clinical Chemistry, Erasmus Medical Center, Wytemaweg 80, 3015 CN Rotterdam, the Netherlands; Department of Internal Medicine, Erasmus Medical Center, Wytemaweg 80, 3015 CN Rotterdam, the Netherlands

## Abstract

**Introduction:**

Children admitted to a pediatric intensive care unit (ICU) are at high risk of developing acute kidney injury (AKI). Although serum creatinine (SCr) levels are used in clinical practice, they are insensitive for early diagnosis of AKI. Urinary neutrophil gelatinase-associated lipocalin (uNGAL) and kidney injury molecule-1 (KIM-1) are novel AKI biomarkers whose performance in pediatric ICU patients is largely unknown. In this study, we aimed to characterize uNGAL and KIM-1 patterns in children following ICU admission and to assess their properties in relation to identifying children at risk for AKI development.

**Methods:**

From June 2010 until January 2014, we conducted a prospective observational cohort study of term-born children ages 1 day to 1 year on mechanical ventilation. Blood and urine samples were obtained every 6 to 12 hours up to 72 hours post-admission. Blood samples were assayed for SCr, and urine samples were assayed for uNGAL and KIM-1. The RIFLE (risk, injury, failure, loss, end-stage renal disease) classification as 150%, 200% or 300% of median SCr reference values was used to define AKI.

**Results:**

A total of 100 children were included (80 survived). Their median age at admission was 27.7 days (interquartile range (IQR), 1.5 to 85.5). The median duration of mechanical ventilation was 5.8 days (IQR, 3.1 to 11.4). Thirty-five patients had evidence of AKI within the first 48 hours post-admission, of whom 24 (69%) already had AKI when they entered the ICU. uNGAL and KIM-1 concentrations in AKI peaked between 6 to 12 hours and between 12 to 24 hours post-admission, respectively. The maximal area under the receiver operating characteristic curve (AUC) for uNGAL was 0.815 (95% confidence interval (CI), 0.685 to 0.945, *P* <0.001) at 0 to 6 hours post-admission. The discriminative ability of KIM-1 was moderate, with a largest AUC of 0.737 (95% CI, 0.628 to 0.847; *P* <0.001) at 12 to 24 hours post-admission. At the optimal cutoff point (126 ng/ml), uNGAL concentration predicted AKI development correctly in 16 (84%) of 19 children, up to 24 hours before a rise in SCr became apparent.

**Conclusions:**

Levels of uNGAL and KIM-1 increase in patients with AKI following ICU admission and peak at 6 to 12 hours and 12 to 24 hours post-admission, respectively. uNGAL seems to be a reliable marker for identifying children who will develop AKI 24 hours later.

## Introduction

Acute kidney injury (AKI) is a frequent and serious complication in critically ill children [1-7]. Moreover, it has been shown to be an independent risk factor for mortality, prolonged length of intensive care unit (ICU) stay and prolonged mechanical ventilation [1,2,5]. Current consensus criteria for diagnosing AKI are based on changes in serum creatinine (SCr) and urine output [4,8]. One must realize, however, that SCr is an indicator of glomerular function rather than renal tubular cell damage, which typically occurs during the initial phase of AKI in ICU patients [9,10]. In addition, a substantial number of functioning nephrons have to be compromised before changes in SCr levels become evident [11]. Moreover, SCr is influenced by factors unrelated to renal function and in the newborn reflects maternal levels immediately after birth [11,12]. Altogether, SCr is increasingly considered a late and not very sensitive marker for diagnosing AKI.

Therefore, research has increasingly focused on the identification of novel, more sensitive biomarkers for renal injury, especially tubular, injury, including urinary neutrophil gelatinase-associated lipocalin (uNGAL) and kidney injury molecule-1 (KIM-1) [13]. NGAL is a small, 25 kDa protein initially discovered in activated human neutrophils [14]. NGAL is expressed in limited quantities in other human tissues, including the lungs, spleen and kidneys, where it is thought to inhibit bacterial growth, scavenge iron and induce epithelial growth [14-18]. Plasma NGAL is freely filtered by the glomerulus and then largely reabsorbed by proximal tubular cells [19]. Upon renal tubular injury, NGAL reabsorption may be decreased, whereas NGAL *de novo* synthesis in epithelial cells of the loop of Henle and of distal tubule segments is strongly upregulated, after which it is found in high concentrations in the urine [20]. KIM-1 is a 104 kDa type I transmembrane glycoprotein that contains both an immunoglobulin-like domain and a mucin domain in its extracellular portion [21]. It is expressed in low levels in healthy proximal tubule cells and is thought to promote apoptotic and necrotic cell clearance [22]. Upon kidney ischemia or toxicity, KIM-1 is highly upregulated and shed into the extracellular space and urine [21,22].

The usefulness of uNGAL was first recognized by Mishra and colleagues, who demonstrated that postoperative uNGAL levels in children at 2 hours after cardiopulmonary bypass (CPB) had a nearly 100% accuracy for predicting AKI at 24 to 72 hours [23]. Subsequent studies, mainly in adults, in clinical settings such as critical care and kidney transplantation confirmed this finding [24,25]. KIM-1, on the other hand, has been systematically investigated only in patients undergoing CPB and in a small sample of asphyxiated neonates [26,27]. Zappitelli and colleagues [28] were the first to evaluate uNGAL in a large, heterogeneous group of pediatric ICU (PICU) patients. They found a good diagnostic marker for development of AKI and persistent AKI for ≥48 hours, but not for AKI if uNGAL had been measured after a rise in SCr [28]. In later studies focused on cutoff points for AKI prediction specifically in PICU patients, researchers evaluated biomarker combinations and even suggested uNGAL as a predictor of mortality [29,30].

None of these previous studies, however, provided insight into biomarker evolution using time intervals based on the hours shortly after ICU admission. Besides, for these studies, authors reported data for subjects with age ranges widely varying from 1 week to 21 years [27-31]. None focused on children up to 1 year of age, even though this age group is particularly vulnerable to renal injury during the physiological evolution of renal function. Therefore, our aim in the present study was to characterize temporary uNGAL and KIM-1 patterns in the 3 days following ICU admission in a large cohort of critically ill children up to 1 year of age. Secondarily, we aimed to assess whether the levels of these biomarkers during the first 24 hours of PICU admission can reliably identify children at risk for AKI development within 48 hours following admission.

## Material and methods

### Setting

From June 2010 until January 2014, a single-center prospective observational cohort study was conducted in the level III ICU of the Erasmus Medical Center–Sophia Children’s Hospital, Rotterdam, the Netherlands. Considered for enrollment were children (born later than 37 weeks of gestational age) between the ages of 1 day and 1 year admitted to the ICU and requiring endotracheal intubation and mechanical ventilation. Patients were not eligible for inclusion if (1) they had congenital abnormalities of the kidney or urinary tract, (2) death was anticipated within 24 hours or (3) they received mechanical ventilation for other reasons (for example, neuromuscular disease). Patients were excluded when treatment with extracorporeal membrane oxygenation was required within the study period. The study protocol was approved by the local medical ethics review board of the Erasmus Medical Center. A deferred consent process was used whereby written informed consent was obtained from the primary caregivers within 12 hours following the study start. The collected blood and urine specimens of those children for whom consent was withdrawn were destroyed (n = 10 children, maximum of 1.4 ml of blood per patient).

### Sample collection and analytical procedures

Upon ICU admission, blood and urine samples were prospectively collected concurrently during the patient’s admission between 0 and 6 hours (T0), 6 and 12 hours (T1), 12 and 24 hours (T2), 24 and 36 hours (T3), 36 and 48 hours (T4) and 48 and 72 hours (T5). For each time frame, 0.7 ml of blood was drawn with an indwelling arterial line, if available, or by capillary or venous puncture. Urine samples were collected using a bladder catheter. To collect 3 ml of freshly voided urine, the urine collection bag was emptied 1 hour prior to each sampling time frame. Urine samples were left refrigerated for sedimentation for 2 to 3 hours, aliquoted and stored within 4 hours after collection at −80°C until performing the assay.

Creatinine concentrations were measured in the hospital’s clinical chemical laboratory by using an enzymatic assay (Creatinine Plus; Roche Diagnostics, Branchburg, NJ, USA) on a cobas 8000 analyzer (Roche Diagnostics). During the period of sample collection, the interassay coefficient of variation (CV) was less than 2.6%.

uNGAL was measured using the latest uNGAL chemiluminescence microparticle immunoassay developed for a standardized clinical platform (ARCHITECT immunoassay analyzer; Abbott Diagnostics Division, Abbott Laboratories, Abbott Park, IL, USA). The mean interassay CV for uNGAL was 5.3% at a concentration of 19.4 ng/ml. The limit of quantification (LoQ) of uNGAL was 3.0 ng/ml, and the upper limit of quantitation was 6,000 ng/ml. The reagents and calibrator for the uNGAL assays were kindly supplied by Abbott Diagnostics. KIM-1 was measured using a commercially available enzyme-linked immunosorbent assay kit (BioAssay Works, Ijamsville, MD, USA). The mean interassay CV for KIM-1 was <14% at a concentration of 0.17 ng/ml. The LoQ of KIM-1 was 0.08 ng/ml. uNGAL and KIM-1 levels are expressed in absolute values (nanograms per milliliter).

### Data collection

Recorded patient data included clinical characteristics and results of laboratory tests. More specifically, sex, gestational age and birth weight, as well as age, body weight and diagnosis at admission, were collected. The Pediatric Risk of Mortality II (PRISM II) score and Pediatric Index of Mortality 2 score were collected as an indication of severity of illness [32,33]. Furthermore, cardiac arrest upon ICU admission and type of mechanical ventilation were registered, together with the fraction of inspired oxygen at the time of intubation, the need for nitric oxide ventilation, and the administration of vasopressor drugs, diuretics (furosemide or bumetanide) or aminoglycosides (gentamicin, tobramycin or amikacin). Last, data on the following outcomes were collected: treatment with renal replacement therapy, duration of mechanical ventilation, lengths of ICU and hospital stay and survival until ICU discharge.

### Definitions

AKI was defined according to the maximal SCr-based RIFLE (risk (R), injury (I), failure (F), loss, end-stage renal disease) score obtained within the first 48 hours following admission. The RIFLE classifications we used defined three grades of increasing AKI severity, including R (risk for kidney injury), I (injury to the kidney) and F (failure of kidney function) [8]. RIFLE outcome categories L (loss of renal function) and E (end-stage renal disease) were not applicable, as the study was restricted to the first 7 days following ICU admission. Because all children enrolled were younger than 1 year of age, most did not have data available for baseline SCr concentrations pre-ICU admission, nor is there an algorithm available to calculate estimated glomerular filtration rate, which is why the RIFLE categories risk, injury and failure were defined as SCr concentrations above 150%, 200% and 300%, respectively, of the median age-specific SCr reference value. These SCr reference values were obtained from a large cohort of children without kidney disease by using short age intervals ranging from 1 day in the first week after birth up to 3 months at the end of the first year of age [34]. *Persistent AKI* was defined as lack of improvement of RIFLE score within 72 hours post-admission.

### Statistical analysis

Unless indicated otherwise, continuous data are expressed as median values with interquartile ranges (IQRs) and discrete data as numbers with percentages. Patients were grouped according to whether they lacked AKI or had AKI (based on RIFLE score for risk, injury and/or failure) within 48 hours following admission. Clinical characteristics and biomarker levels were compared between AKI and non-AKI patients using univariate analyses for continuous variables (Mann–Whitney *U* test) and categorical variables (Pearson’s χ^2^ test or Fisher’s exact test, as appropriate). Biomarker levels were also compared between RIFLE strata and diagnostic categories using univariate overall comparisons between groups (Kruskal-Wallis test). Receiver operating characteristic (ROC) curves were generated for the occurrence of AKI within 48 hours following intubation using biomarker levels within three different time frames (T0, T1 and T2: 0 to 6 hours, 6 to 12 hours and 12 to 24 hours, respectively) as well as 24-hour peak levels. The areas under the ROC curve (AUCs) with 95% confidence intervals (95% CI) were calculated. Also, for each time frame, the optimal cutoff value based on the Youden index was calculated with corresponding sensitivity and specificity. Using those cutoff values, sensitivity and specificity of both biomarkers for predicting AKI, as well as the positive and negative predictive values, were calculated for patients who developed AKI later within the study period (<72 hours post-admission) after being considered AKI-free upon admission. Of these patients, the timing and absolute values of maximum biomarker levels in urine samples preceding AKI were compared with biomarker levels in the first urine samples of controls (critically ill children who did not have AKI). A two-sided *P*-value of 0.05 was considered the limit of significance in all analyses. Data were analyzed using IBM SPSS Statistics version 21 software (IBM, Armonk, NY, USA).

## Results

### Patients

A total of 110 patients were initially included by deferred consent. However, because consent was withdrawn by 10 (9%) parents, 100 patients were ultimately enrolled in the study. Table 1 details the characteristics of all patients stratified by occurrence of AKI within 48 hours post-admission. At admission, median age was 27.7 days (IQR, 1.5 to 85.5), and median body weight was 3.8 kg (IQR, 3.2 to 5.3). The most common primary diagnoses were congenital diaphragmatic hernia (n = 23 (23%)) and respiratory failure (n = 20 (20%)). For all patients, the median duration of mechanical ventilation was 5.8 days (IQR, 3.1 to 11.4), and the median ICU stay was 10.0 days (IQR, 6.1 to 27.0). Seventeen patients (17%) died during admission after a median ICU stay of 11.7 days (IQR, 4.4 to 27.2).

**Table 1:** **Patient characteristics grouped according to occurrence of acute kidney injury** ^**a**^

	**All patients (n = 100)**	**Non-AKI (n = 65)**	**AKI (n = 35)**	***P*** **-value** ^**b**^
Baseline characteristics				
Male sex	66 (66)	42 (65)	24 (69)	0.690^c^
Gestational age, wk	39.0 (37.6 to 40.0)	38.9 (37.8 to 40.0)	39.0 (37.4 to 40.0)	0.861^d^
Birth weight, kg	3.1 (2.8 to 3.6)	3.1 (2.8 to 3.5)	3.1 (2.8 to 3.6)	0.989^d^
Clinical characteristics at intubation				
Age, days	27.7 (1.5 to 85.5)	27.1 (1.8 to 71.4)	30.3 (1.4 to 115.0)	0.667^d^
Weight, kg	3.8 (3.2 to 5.2)	3.7 (3.3 to 5.0)	3.8 (3.1- 6.0)	0.745^d^
Admission diagnosis				
Congenital diaphragmatic hernia	23 (23)	16 (25)	7 (20)	0.132^c^
Respiratory failure	20 (20)	11 (17)	9 (26)	
Cardiac failure	18 (18)	8 (12)	10 (28)	
RSV bronchiolitis	17 (17)	14 (21)	3 (9)	
Sepsis	14 (14)	9 (14)	5 (14)	
Other	8 (8)	7 (11)	1 (3)	
Cardiac arrest on ICU admission, yes	10 (10)	5 (8)	5 (14)	0.283^c^
Severity of illness at ICU admission				
PIM2, %	9.8 (3.4 to 18.8)	7.0 (1.7 to 12.0)	15.9 (8.4 to 38.1)	0.011^d^
PRISM II, %	33.8 (11.0 to 64.5)	25.7 (10.1 to 54.1)	48.9 (25.5 to 77.1)	<0.001^d^
Type of mechanical ventilation				
Pressure control	68 (68)	45 (69)	23 (66)	0.671^c^
Pressure-regulated volume control	19 (19)	11 (17)	8 (23)	
High-frequency ventilation	11 (11)	7 (11)	4 (11)	
Pressure support	2 (2)	2 (3)	–	
Fraction of inspired oxygen at intubation, percentage	59 (40 to 94)	55 (40 to 90)	68 (39 to 100)	0.422^d^
Need for nitric oxide ventilation at intubation, yes	18 (18)	10 (15)	8 (23)	0.354^c^
Need for two or more vasopressors at intubation, yes	49 (49)	24 (37)	25 (71)	0.001^c^
Diuretic drugs, yes	77 (76)	46 (71)	30 (86)	0.095^d^
Aminoglycosides, yes	37 (37)	24 (36)	13 (37)	0.983^d^
Outcomes				
Need for renal replacement therapy, yes	1 (1)	–	1 (3)	N/A
Duration of mechanical ventilation, days	5.8 (3.1 to 11.4)	4.4 (3.0 to 8.3)	8.3 (5.6 to 19.1)	0.001^d^
Length of ICU stay, days	10.0 (6.1 to 27.0)	8.3 (5.8 to 15.9)	19.2 (7.8 to 35.6)	0.002^d^
Length of hospital stay, days	15.9 (8.1 to 38.0)	11.6 (6.5 to 27.9)	27.0 (11.1 to 46.9)	0.015^d^
Mortality	20 (20)	9 (14)	11 (32)	0.027^c^
ICU	17	7	10	N/A
Time from admission until death, days	11.7 (4.4 to 27.2)	9.9 (3.4 to 17.0)	18.7 (6.7 to 45.0)	N/A

^a^AKI, Acute kidney injury; ICU, Intensive care unit; N/A, Not applicable; PIM2, Pediatric Index of Mortality 2; PRISM II, Pediatric Risk of Mortality II; RSV, Respiratory syncytial virus. Patient demographic data and clinical characteristics of all patients enrolled, grouped according to the development of AKI (yes or no), are shown. AKI was defined according to the highest RIFLE (risk, injury, failure, loss, end-stage renal disease) score attained within 48 hours following admission. The RIFLE categories risk, injury and failure were defined as, respectively, serum creatinine (SCr) above 150%, 200% and 300% of the median age-specific SCr reference values. Continuous data are expressed as median (interquartile range), and categorical data are expressed as number (%). ^b^ *P*-values indicate comparison between AKI and non-AKI patients using univariate analyses. ^c^Pearson’s χ^2^ test or Fisher’s exact test, as appropriate, for categorical variables. ^d^Mann–Whitney *U* test for continuous variables.

### Acute kidney injury

Thirty-five patients (35%) met the criteria for AKI within 48 hours following ICU admission. Fifteen (42%) of those were classified as RIFLE-R, 10 (29%) as RIFLE-I and 10 (29%) as RIFLE-F. One patient classified as RIFLE-F received renal replacement therapy starting 2 days post-admission. Twenty-four (69%) of the thirty-five patients with AKI already met the criteria for AKI at the time of admission (risk: n = 6 (25%); injury: n = 8 (33%); failure: n = 10 (42%)).

Age, weight, diagnosis and cardiac arrest experienced upon ICU admission did not significantly differ between patients who developed AKI within 48 hours following admission and those who did not (Table 1). Nonetheless, patients in the former group were more severely ill on admission, as reflected by a significantly higher PRISM II score (48.9 (IQR, 25.5 to 77.1)) than that assigned to non-AKI patients (25.1 (IQR, 10.10 to 54.1) (*P* <0.001 by Mann–Whitney *U* test). Moreover, patients with AKI were ventilated almost twice as long as non-AKI patients, although type of mechanical ventilation, fraction of inspired oxygen and need for nitric oxide ventilation did not differ. Patients with AKI more often received two or more types of vasopressor drugs at intubation and had longer ICU and hospital lengths of stay. In contrast, there was no difference in the prescription of diuretic drugs and aminoglycosides between AKI and non-AKI patients. Last, the overall mortality rate in patients with AKI was significantly higher than that of non-AKI patients (32% versus 14%; *P* = 0.027 by Pearson’s χ^2^ test).

### Biomarker patterns post-admission

In total, 491 urine samples were collected (that is, 86% of all scheduled samples (5 samples (IQR, 4 to 5) per patient). Sampling was not feasible in cases of anuria, discontinuation of bladder catheterization or logistical problems. The median uNGAL concentration for all urine specimens was 39.5 ng/ml (IQR, 12.4 to 168.1 ng/ml) and the median KIM-1 concentration was 0.14 ng/ml (IQR, 0.08 to 0.30 ng/ml). For patients with AKI, the median uNGAL and KIM-1 concentrations were 107 ng/ml (IQR, 22.4 to 935) and 0.19 ng/ml (IQR, 0.10 to 0.43), respectively, which were significantly higher than those for non-AKI patients: uNGAL of 23.2 ng/ml (IQR, 9.6 to 93) and KIM-1 of 0.13 ng/ml (IQR, 0.08 to 0.25) (both *P*-values <0.001 by Mann–Whitney *U* test).

**Figure 1:**
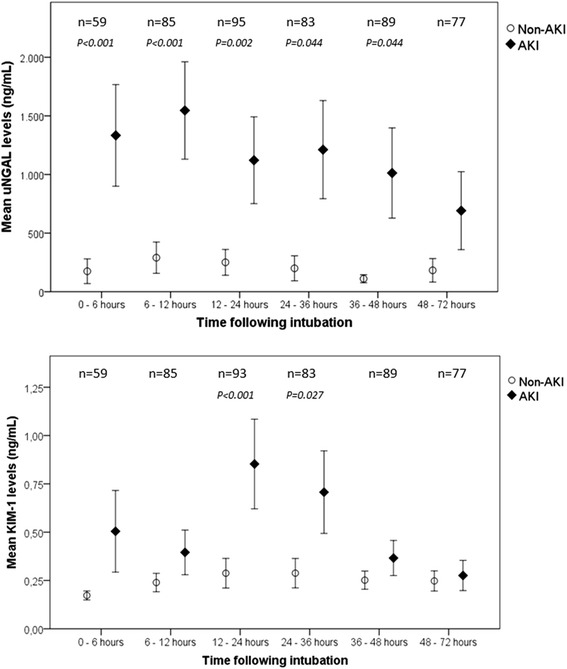
Clinical course of mean urinary neutrophil gelatinase-associated lipocalin and kidney injury molecule**-**1 levels from 0 to 72 hours following intubation, stratified by occurrence of acute kidney injury within 48 hours post-admission. Data represent the mean (±1 standard error of the mean). The filled diamonds represent patients with acute kidney injury (AKI), and the open circles represent non-AKI patients. Differences were assessed for each biomarker per time frame using Mann–Whitney *U* tests. KIM-1, Kidney injury molecule-1; uNGAL, Urinary neutrophil gelatinase-associated lipocalin.

**Figure 2:**
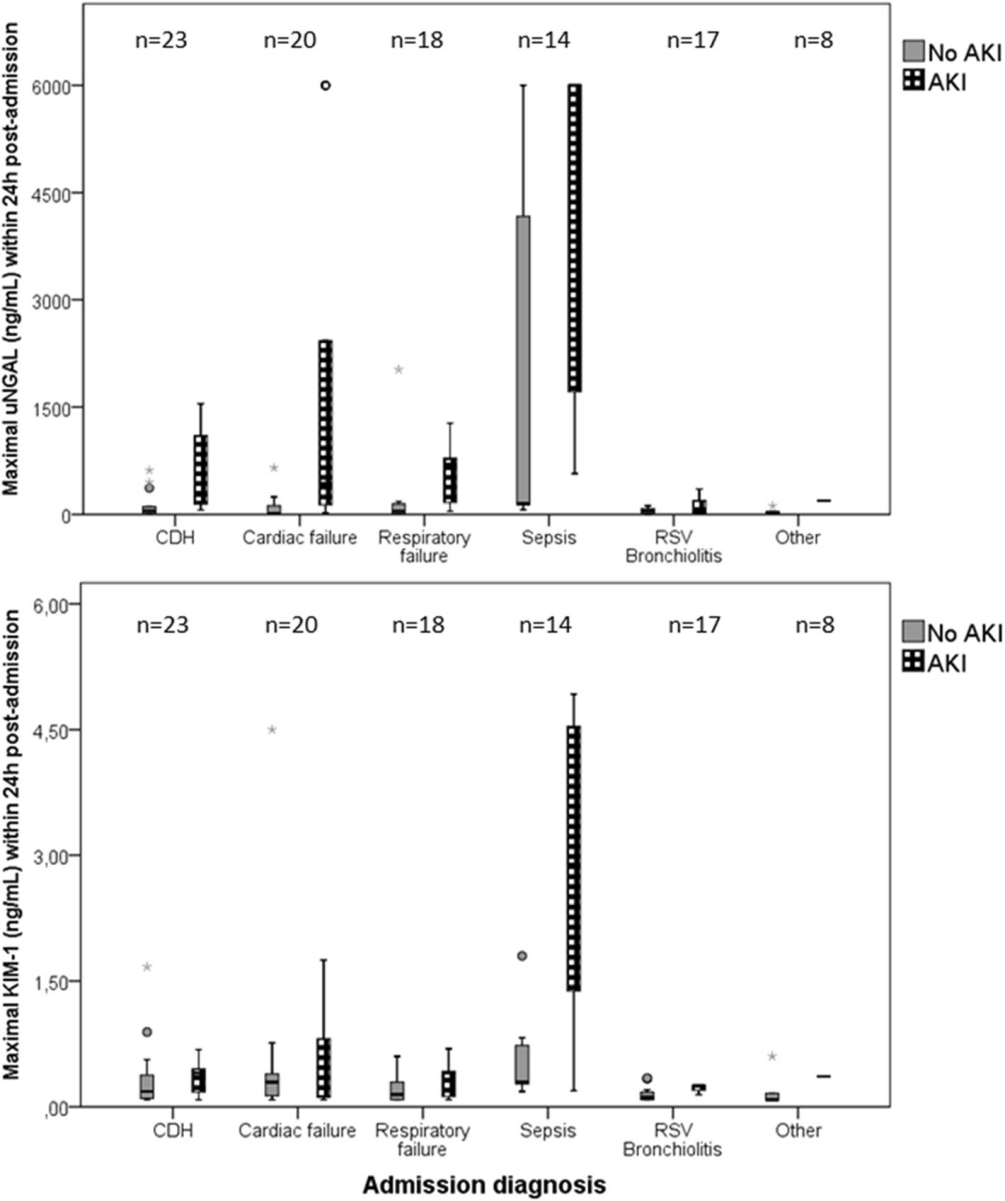
Maximal urinary neutrophil gelatinase-associated lipocalin and kidney injury molecule-1 levels within 24 hours post-admission, stratified by diagnosis upon admission (total number of patients = 100). Data represent the mean (±1 standard error of the mean). The black-and-white boxes represent patients with acute kidney injury (AKI), and the gray boxes represent non-AKI patients. Intergroup differences were assessed using Kruskal-Wallis tests. CDH, Congenital diaphragmatic hernia; KIM-1, Kidney injury molecule-1; RSV, Respiratory syncytial virus; uNGAL, Urinary neutrophil gelatinase-associated lipocalin.

Figure 1 presents the patterns of median uNGAL and KIM-1 levels from 0 to 72 hours following ICU admission. Both patterns showed an increase; the mean uNGAL concentration peaked at 6 to 12 hours, and the mean KIM-1 concentration peaked at 12 to 24 hours. uNGAL levels were significantly higher in the patients who developed AKI than in the non-AKI patients at all time frames except for T6 (48 to 72 hours) (all *P*-values ≤0.038 by Mann–Whitney *U* test). KIM-1 levels were significantly higher in patients with AKI only at T2 (12 to 24 hours) and T3 (24 to 36 hours) (both *P*-values ≤0.041 by Mann–Whitney *U* test). Table 2 shows the uNGAL and KIM-1 concentrations for T0 (0 to 6 hours), T1 (6 to 12 hours) and T2 (12 to 24 hours) and the peak levels within 24 hours by SCr-based RIFLE strata. Worse RIFLE status was significantly associated with higher uNGAL and KIM-1 levels (all *P*-values ≤0.018 by Kruskal-Wallis test). Compared across diagnostic categories, uNGAL and KIM-1 concentrations were both highest in patients with sepsis, especially those who met the AKI criteria (both *P*-values <0.001 by Kruskal-Wallis test) (Figure 2).

**Table 2:** **T0, T1, T2 and peak urinary neutrophil gelatinase-associated lipocalin and kidney injury molecule-1 concentrations by serum creatinine-based RIFLE status within 48 hours following admission** ^**a**^

**Measurement, number of patients**	**Non-AKI, n = 65**	**RIFLE-Risk, n = 15**	**RIFLE-Injury, n = 10**	**RIFLE-Failure, n = 10**	***P*** **-value**
T0, 0 to 6 hr					
uNGAL (ng/ml), n = 59	31 (16 to 111)	396 (70to 1,250)	385 (77 to 1,987)	1,873 (484 to 3,936)	<0.001
KIM-1 (ng/ml), n = 59	0.11 (0.08 to 0.19)	0.18 (0.13 to 0.28)	0.10 (0.08 to 0.11)	1.2 (0.4 to 3.5)	0.004
T1, 6 to 12 hr					
uNGAL (ng/ml), n = 85	21 (8 to 116)	114 (61 to 420)	275 (11 to 4630)	2430 (727 to 6000)	<0.001
KIM-1 (ng/ml), n = 85	0.11 (0.08 to 0.27)	0.10 (0.08 to 0.29)	0.12 (0.08 to 0.16)	0.35 (0.29 to 1.21)	0.018
T2, 12 to 24 hr					
uNGAL (ng/ml), n = 95	22 (10 to 98)	34 (22 to 200)	47 (26 to 1935)	979 (301 to 6000)	0.001
KIM-1 (ng/ml), n = 93	0.16 (0.08 to 0.28)	0.26 (0.11 to 0.56)	0.30 (0.12 to 0.41)	0.47 (0.26 to 2.15)	0.002
24-hr peak level					
uNGAL (ng/ml), n = 100	59 (16 to 136)	225 (89 to 730)	385 (56 to 3938)	1495 (387 to 6000)	<0.001
KIM-1 (ng/ml), n = 100	0.17 (0.08 to 0.34)	0.26 (0.11 to 0.56)	0.25 (0.10 to 0.41)	0.86 (0.44 to 2.15)	0.001

^a^AKI, Acute kidney injury; KIM-1, Kidney injury molecule-1; RIFLE, Risk, injury, failure, loss, end-stage renal disease; SCr, Serum creatinine; uNGAL, Urinary neutrophil gelatinase-associated lipocalin. Data are expressed as median (interquartile range). *P*-values indicate overall comparison of all groups (that is, non-AKI versus Risk versus Injury versus Failure). Intergroup differences were assessed using the Kruskal-Wallis test. RIFLE categories risk, injury and failure were defined as, respectively, serum creatinine (SCr) above 150%, 200% and 300% of the median age-specific SCr reference values.

### Urinary neutrophil gelatinase-associated lipocalin, kidney injury molecule-1 and mortality

Seventeen patients died during admission. Three of these non-survivors died within the first 72 hours, of whom one was classified in the non-AKI group (died after 66 hours, peak uNGAL value of 114 ng/ml) and two were in the AKI-group (died after 46 and 54 hours, respectively, both with peak uNGAL values of 6,000 ng/ml). Non-survivors’ uNGAL levels at 12 to 24 hours post-admission, as well as their 24-hour peak levels, were higher than those of survivors (*P*-values ≤0.009 by Mann–Whitney *U* test). There was no significant difference between survivors and non-survivors with regard to KIM-1 levels (all *P*-values >0.05).

### Area under the receiver operating characteristic curve analysis

**Figure 3:**
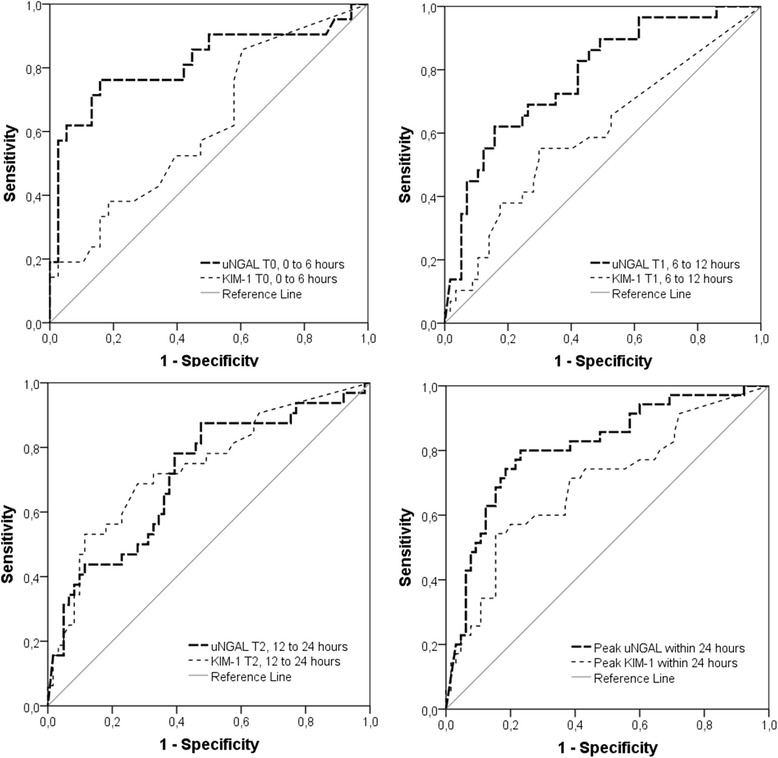
Receiver operating characteristic curves of urinary neutrophil gelatinase-associated lipocalin and kidney injury molecule-1 levels at time frames T0, T1 and T2 and for the 24-hour peak levels. The dashed black lines represent the receiver operating characteristic (ROC) curves of urinary neutrophil gelatinase-associated lipocalin (uNGAL), and the dotted lines represent the ROC curves of kidney injury molecule-1 (KIM-1). The gray lines represent the reference value.

Table 3 shows the AUCs for the prediction of the development of AKI within 48 hours following admission for both biomarkers at T0, T1 and T2, as well as for 24-hour peak levels. The maximal AUC for uNGAL was 0.815 (95% CI, 0.685 to 0.945; *P*-value <0.001) at T0 (0 to 6 hours), with an optimal cutoff value of 126 ng/ml with a sensitivity of 76% and a specificity of 84%. The AUCs for uNGAL were 0.780 (95% CI, 0.678 to 0.882; *P*-value <0.001) at T1, 0.711 (95% CI, 0.599 to 0.824; *P*-value =0.001) at T2 and 0.811 (95% CI, 0.719 to 0.902; *P*-value <0.001) for the 24-hour peak levels. KIM-1 was moderately discriminative only at T2, with an AUC of 0.737 (95% CI, 0.628 to 0.847; *P*-value <0.001) and an optimal cutoff value of 0.19 ng/ml with a sensitivity of 72% and specificity of 67%. Figure 3 shows the ROC curve of uNGAL and KIM-1 levels at T0, T1 and T2 and for the 24-hour peak levels.

**Table 3:** **Occurrence of acute kidney injury within 48 hours following intubation** ^**a**^

**Biomarker**	**Number of patients (%)**	**Time frame**	**AUC (95% CI)**	***P*** **-value**	**Cutoff (ng/ml)**	**Sensitivity (%)**	**Specificity (%)**
uNGAL	59 (59%)	T0, 0 to 6 hr	0.815 (0.685 to 0.945)	<0.001	126	76	84
	85 (85%)	T1, 6 to 12 hr	0.780 (0.678 to 0.882)	<0.001	88	70	74
	95 (95%)	T2, 12 to 24 hr	0.711 (0.599 to 0.824)	0.001	32	72	62
	100 (100%)	24-hr peak level	0.811 (0.719 to 0.902)	<0.001	1,338	80	77
KIM-1	58 (57.4%)	T0, 0 to 6 hr	0.618 (0.469 to 0.768)	0.135	0.15	52	60
	85 (85%)	T1, 6 to 12 hr	0.553 (0.469 to 0.729)	0.135	0.13	55	60
	93 (931%)	T2, 12 to 24 hr	0.737 (0.628 to 0.847)	<0.001	0.19	72	67
	100 (100%)	24-hr peak level	0.695 (0.584 to 0.807)	0.001	0.24	71	62

^a^AUC, Area under the receiver operating characteristic curve; CI, Confidence interval; KIM-1, Kidney injury molecule-1; uNGAL, Urinary neutrophil gelatinase-associated lipocalin. The optimal cutoff was based on the Youden index.

### Urinary neutrophil gelatinase-associated lipocalin and kidney injury molecule-1 concentrations preceding acute kidney injury

**Figure 4:**
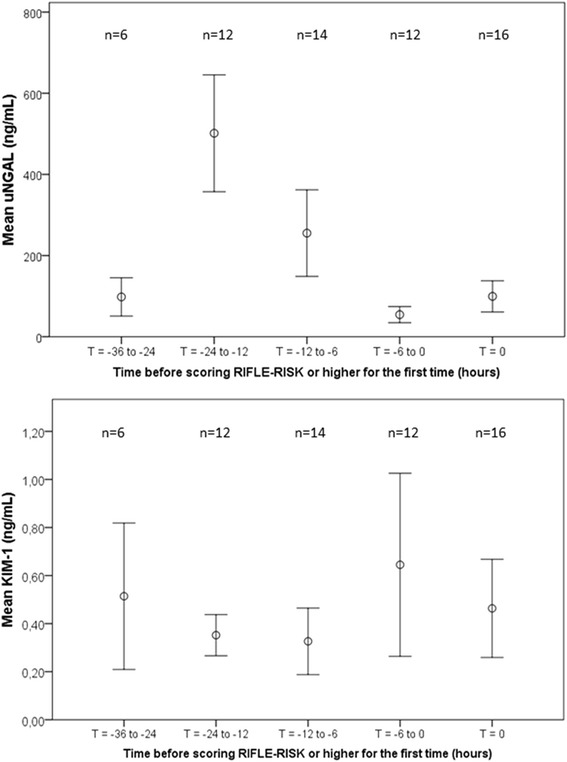
Urinary neutrophil gelatinase-associated lipocalin and kidney injury molecule-1 levels prior to the onset of acute kidney injury, defined as attaining RIFLE-Risk level or higher (n = 19). Data represent the mean (±1 standard error of the mean). Acute kidney injury (AKI) was defined as a rise in serum creatinine of 150% or greater compared with age-specific reference values, which equals RIFLE-Risk level or higher. KIM-1, Kidney injury molecule-1; RIFLE, Risk, injury, failure, loss, end-stage renal disease; uNGAL, Urinary neutrophil gelatinase-associated lipocalin.

Twenty-four patients already met the AKI criteria at the time of ICU admission, and nineteen other patients developed AKI later within the 72-hour admission (eleven within 48 hours and another eight between 48 and 72 hours post-admission). These 19 patients reached RIFLE-Risk level or higher at a median of 34 hours (IQR, 20 to 53) post-admission (Risk: n = 16 (84%); Injury: n = 3 (16%)). For the analysis of biomarkers preceding AKI, the time point at which AKI first occurred was recoded to T0. All available uNGAL and KIM-1 measurements preceding this time point were recoded relative to T0 (Figure 4). Using the optimal cutoff value for uNGAL (126 ng/ml) and KIM-1 (0.19 ng/ml) levels as described above, uNGAL was the most sensitive biomarker for predicting the development of AKI (that is, in 16 (84%) of 19 cases using uNGAL concentration versus 11 (58%) of 19 cases using KIM-1 concentration).

**Figure 5:**
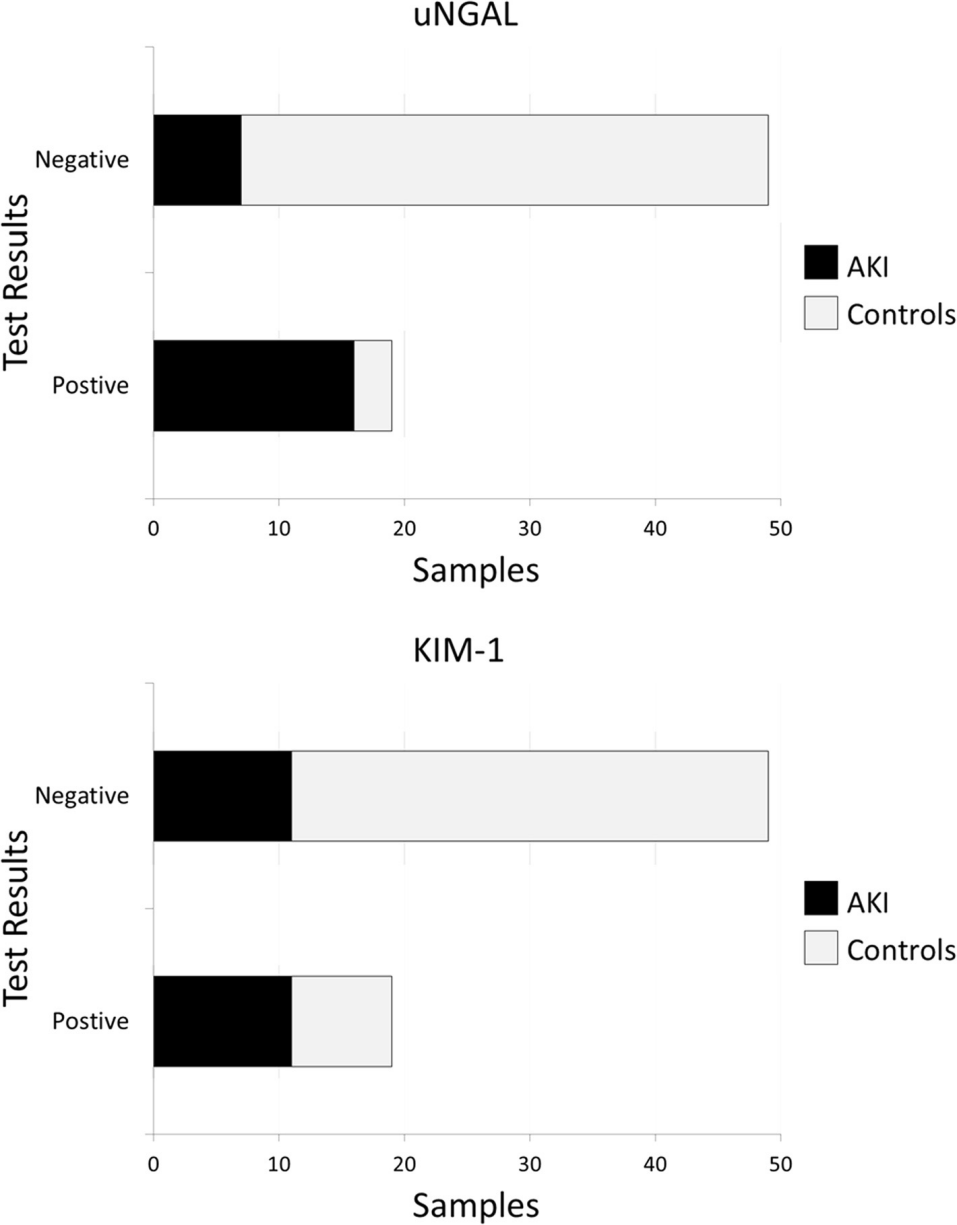
Results of contingency analysis. Bar graphs show the results of a contingency table analysis for urinary neutrophil gelatinase-associated lipocalin (uNGAL; cutoff value = 126 ng/ml) and kidney injury molecule-1 (KIM-1; cutoff value of 0.19 ng/ml). A total of 49 control samples were included (non-AKI critically ill), as well as 19 from critically ill children who developed AKI within 72 hours post-admission. AKI, Acute kidney injury.

The maximum biomarker levels in urine samples preceding AKI (T0) were then used to evaluate the diagnostic performances of both biomarkers for AKI prediction. The first urine samples of 49 patients who did not develop AKI served as controls. The clinical characteristics and outcomes of these 49 control patients did not differ from those of the 19 patients with AKI (all *P*-values >0.05). Maximum uNGAL and KIM-1 levels were observed at medians of 22 hours (IQR, 12 to 24) and 9 hours (IQR, 5 to 15), respectively, before reaching RIFLE-Risk level or higher for the first time. A contingency table analysis using a cutoff value >126 ng/ml for uNGAL showed that the sensitivity of uNGAL was 84%, its specificity was 86%, its positive predictive value was 70% and its negative predictive value was 93% (Figure 5). At a cutoff value of 0.19 ng/ml, the sensitivity of KIM-1 was 58%, its specificity was 78%, its positive predictive value was 50% and its negative predictive value was 83%.

### Persistent acute kidney injury

Of the 24 patients who had AKI upon admission, 13 had persistent AKI in the time frame between 48 and 72 hours, whereas the RIFLE score improved in 11. Biomarker levels did not differ significantly between groups.

## Discussion

The aim of this study was to characterize uNGAL and KIM-1 patterns throughout the first days of ICU admission in critically ill children requiring mechanical ventilation and to assess their properties for identifying children who develop AKI. We show that levels of both biomarkers increased following admission and were significantly higher with worsening AKI severity. Most important, we found that uNGAL levels in the first 6 hours following admission can serve as a marker for identifying children meeting AKI criteria within 48 hours of ICU admission. Besides, when using the optimal cutoff uNGAL value (126 ng/ml), 16 of 19 patients in whom AKI was detected 24 hours later would have been diagnosed correctly. In contrast, KIM-1 was not found to be reliable in identifying children at risk for AKI. Thus, in a heterogeneous group of critically ill children, uNGAL may allow for an early diagnosis of AKI, even before a rise in SCr becomes apparent.

Of the 35 patients diagnosed with AKI within 48 hours following admission, a remarkable proportion of almost 70% already had AKI upon admission, including the most severe cases. These figures are in line with a large retrospective cohort study in which investigators evaluated 2,106 admissions to the PICU in whom AKI occurred predominantly on the first PICU day [2].

Overall, we found notably higher uNGAL and KIM-1 biomarker levels than reported previously [27,28]. Zappitelli and colleagues reported a median peak uNGAL level of 55 ng/ml (IQR, 105) for patients with RIFLE-F, whereas we found a median value of 1,495 ng/ml (IQR, 387 to 6,000) [28]. This is even more surprising in light of the fact that 23% of patients in the Zappitelli study had sepsis, compared with only 14% in our study. Our present study as well as other studies have shown that patients with sepsis have the highest levels of uNGAL and KIM-1, irrespective of AKI development, which is at least in part due to systemic inflammation [25,31,35,36]. Another explanation may lie in the lower severity of illness in the Zappitelli cohort, as reflected by a median PRISM II score of 19 (IQR, 12) [28] for the renal failure group compared with 48.9 (IQR, 25.5 to 77.1) in our patients with AKI. Furthermore, as uNGAL and KIM-1 levels in premature infants and young children generally are higher than healthy infants and young children, the younger age of our subjects might form another explanation [29,37,38]. Last, Zappitelli and colleagues used other biomarker kits [28].

We have shown that levels of both biomarkers increased following admission, with uNGAL levels peaking between 6 and 12 hours and KIM-1 levels peaking somewhat later, between 12 and 24 hours. These patterns resemble those reported in a well-conducted study of 543 adult ICU patients in which uNGAL levels in patients with AKI increased starting from the time of admission (*P* <0.0001) and KIM-1 levels first differentiated between non-AKI and AKI patients 24 hours post-admission (*P* = 0.008) [39]. To the best of our knowledge, there are no pediatric studies available on the patterns of biomarkers following ICU admission using hourly time intervals. One study of 13 asphyxiated newborns reported uNGAL and KIM-1 levels on days 1 and 3 of life [27]. Notably, uNGAL levels in that study were increased but remained stable, whereas KIM-1 levels were higher only on the first day of life and substantially decreased thereafter [27].

In our present study, uNGAL measured within 0 to 6 hours following admission had a reasonable ability (ROC-AUC = 0.815) to identify children meeting AKI criteria within 48 hours [25]. The AUC for uNGAL was in perfect line with previous reports in adults (AUCs 0.66 to 0.88, depending on the severity of AKI). The optimal uNGAL cutoff value found (126 ng/ml; sensitivity of 75%, specificity of 84%) was slightly lower than reported for adults (247 ng/ml; sensitivity of 89% and specificity of 70% for prediction of renal failure) [40]. The discriminative ability of KIM-1, in contrast, is only limited, because all AUC values were ≤0.73, which is consistent with previous reports [27,39]. All in all, the most robust NGAL results in critically ill children come from Mishra and colleagues for children who underwent CPB. In their study, AKI timing and etiology post-CPB was well defined [23,25].

Concerning the time relationship between the biomarker levels and AKI development, uNGAL levels in the present study peaked at 12 to 24 hours before reaching RIFLE-Risk level for the first time, whereas KIM-1 levels preceding AKI remained steady. A similar pattern for uNGAL levels was reported in a study of adults, but KIM-1 levels in that study did not rise until the time AKI was diagnosed [39]. Zappitelli and colleagues evaluated uNGAL levels relative to the day of pediatric RIFLE AKI attainment in 21 PICU patients [28]. Although they presented NGAL levels relative to urinary creatinine concentrations, it was clear that median NGAL levels peaked at 1 day before AKI onset [28]. Blood was sampled daily, however, instead of at hourly intervals.

The false-positive results of uNGAL (14%) and KIM-1 (22%) in the present study may be due to the limited specificity of both biomarkers, but one can also speculate that subclinical AKI occurred, a condition in which there is tubular damage without a rise in SCr as a sign of glomerular filtration alteration [10]. Still, uNGAL was able to predict AKI development correctly in 16 of 19 children, which further demonstrates its potential as an early marker of tubular damage. Identifying children in an early stage of AKI (for example, already at presentation in the emergency room or during clinical deterioration at the general ward) may help develop early interventions to limit the development of AKI. In this light, several studies have been published on potential protective properties of drugs against the development of AKI, including atrial natriuretic factor, such as in the cases of cisplatin treatment [41] and cardiac surgery [42], and administration of bovine-derived alkaline phosphatase in critically ill patients with sepsis-associated AKI [43]. Even though the rationale behind the renoprotective effects remains to be fully elucidated, these studies illustrate that new options in the prevention or limitation of the severity of AKI are currently being investigated.

Several limitations of this study should be addressed. First, this study has a single-center design, which may limit the generalization of the data to other institutions. Still, because we did not focus on diagnostic subclasses, our cohort can be considered a representative sample of the general PICU population younger than 1 year of age requiring mechanical ventilation. Second, the overall sample size was too small for multiple subgroup analyses. Besides, because we exclusively used SCr without urinary criteria for grading AKI severity, we may have underestimated the incidence and grade of AKI [44]. Furthermore, we were not able to collect all urine samples planned, especially not during the first hours of admission, when an indwelling urinary catheter yet had to be placed, when the urine portion was needed for clinical purposes (for example screening for metabolic diseases) or when a patient was anuric. Still, 86% of all planned samples were collected. Last, uNGAL and KIM-1 levels preceding AKI could be analyzed in only a small sample because most AKI patients already met RIFLE-Risk criteria or higher upon admission.

## Conclusions

This study shows that both uNGAL and KIM-1 levels in critically ill infants with AKI increase following ICU admission and peak 6 to 12 hours and 12 to 24 hours thereafter, respectively. Notably, of the children who met AKI criteria within 48 hours following admission, almost 70% already had AKI upon admission to the ICU. Still, uNGAL reliably discriminated between infants who met AKI criteria within 48 hours following admission and those who did not. In addition, uNGAL was able to predict AKI development correctly in 84% of children before any rise in SCr became apparent. These findings support the emerging role of uNGAL in identifying AKI at an early stage, which may in the future help us to establish timely renoprotective interventions to reduce AKI in the most vulnerable patients in hospital.

## Key messages

- Approximately 70% of the children who develop AKI within 48 hours of admission already have AKI when entering the ICU.
- In children with AKI, there was a temporal pattern of increase in uNGAL and KIM-1 levels as they peaked between 6 to 12 hours and 12 to 24 hours, respectively, following admission.
- uNGAL concentrations at admission seems to be a reliable marker for identifying children who will develop AKI within 48 hours following admission.

## Abbreviations

AKI: Acute kidney injury
AUC: Area under the receiver operating characteristic curve
CDH: Congenital diaphragmatic hernia
CI: Confidence interval
CKD: Chronic kidney disease
CPB: Cardiopulmonary bypass
CV: Coefficient of variation
ICU: Intensive care unit
IQR: Interquartile range
KIM-1: Kidney injury molecule-1
LoQ: Limit of quantification
PICU: Pediatric intensive care unit
PIM2: Pediatric Index of Mortality 2
PRISM II: Pediatric Risk of Mortality II
RIFLE: Risk, injury, failure, loss, end-stage renal disease
ROC: Receiver operating characteristic
RSV: Respiratory syncytial virus
SCr: Serum creatinine
uNGAL: Urinary neutrophil gelatinase-associated lipocalin
